# Supplementary material for: Cryogenic multiplexing using selective area grown nanowires
Source: Nat Commun. 2023 Nov 25;14:7738. doi: 10.1038/s41467-023-43551-1 (PMC10676361; doi:10.1038/s41467-023-43551-1)
Supplement: Supplementary file 1 — Supplementary Information [file 41467_2023_43551_MOESM1_ESM.pdf]

Supplementary Information
Cryogenic Multiplexing Using Selective Area Grown Nanowires

Dags Olsteins,[1] Gunjan Nagda,[1] Damon J. Carrad,[2] Daria V. Beznasyuk,[2] Christian E. N. Petersen,[2] Sara Martí-Sánchez,[3] Jordi Arbiol,[3,4] and Thomas Sand Jespersen[1,2]

[1] *Center For Quantum Devices, Niels Bohr Institute, University of Copenhagen, 2100 Copenhagen, Denmark*
[2] *Department of Energy Conversion and Storage, Technical University of Denmark, 2800 Kgs.Lyngby, Denmark*
[3] *Catalan Institute of Nanoscience and Nanotechnology (ICN2), CSIC and BIST, Campus UAB, Bellaterra, Barcelona, Catalonia, Spain*
[4] *ICREA, Passeig de Lluís Companys 23, 08010 Barcelona, Catalonia, Spain*

S1. DETAILS OF SELECTIVE AREA GROWTH

The details of the selective area growth (SAG) using MBE are provided here. After preparing the substrate for SAG, the sample is introduced to the MBE system. The sample was degassed in the loadlock for 4 hours at 200° C and then transferred to a second chamber connecting the loadlock to the main chamber. Here, the sample was further degassed at a heating station for 1 hour at 400° C. After transfer to the MBE growth chamber, the substrate was thermally annealed by increasing the substrate temperature to $T_{\mathrm{sub}} = 400°$ C with a ramp rate of 20° C/min and then further to $T_{\mathrm{sub}} = 610°$ C with a ramp rate of 10° C/min under a constant As_4 beam equivalent over-pressure of 1.4×10^{-5} mbar. RHEED was used to monitor the removal of the native oxide at this temperature for approximately 13 minutes (this time varies slightly from sample to sample), the last minute of which the intensity of the specular RHEED spot does not increase[1]. Subsequently, T_{sub} was reduced to the GaAs(Sb) buffer growth temperature of $T_{\mathrm{sub,GaAs(Sb)}} = 600°$ C. For the buffer growth, a As/Ga ratio of 9 and Sb/Ga ratio of 3 was used. This ensures that the growth rate is dependent on the Ga flux and the growth is performed with a Ga growth rate of 0.1 ML/s for 30 min. The substrate temperature was further reduced to $T_{\mathrm{sub,InAs}} = 500°$ C and stabilized during 10 min for InAs growth. An As/In ratio of 9 and an In growth rate of 0.06 ML/s was used and InAs was grown for 18 min. The choice of a lower substrate temperature and a slower growth rate is employed to minimize temperature assisted Ga diffusion into the InAs channel[2]. The substrate temperature was monitored before, shortly at the beginning and after the growth using a pyrometer and the variation in T_{sub} was maximally 2° C. This uncertainty is attributed to the variation in temperature read-out caused by radiation from different regions of the substrate.

S2. CRYSTAL STRUCTURE, STRAIN AND COMPOSITION

Figure S1 shows the morphology, strain and crystal structure of the NWs comparable to NWs investigated in this study. A uniform morphology is visible for 4 InAs/GaAs(Sb) NWs oriented along the $[0\,\bar{1}\,1]$ direction on a GaAs(3 1 1)A substrate in Fig S1a. The InAs channel exhibits $\{1\,1\,1\}$A facets. It should be noted that the cross-sectional shape is specific to the choice of substrate, in-plane orientation, mask dimensions such as the width and pitch, as well as the growth parameters listed in the previous section.

Figure S1b shows the crystal structure of all 4 NWs, along with the corresponding geometric phase analysis (GPA) of the dilatation and rotation maps. Figure S1b (column 2) shows stacking faults (highlighted as white dashed lines) originating at the GaAs(Sb)/InAs interface and propagating towards the InAs channel. It can be observed the dilatation maps in Fig. S1b (column 3, 4) that the main relaxation occurs through creation of an array of dislocations at the GaAs(Sb)/InAs interface. The rotational maps in Fig. S1b (column 5, 6) show rotation in the crystal planes which comes along as an elastic relaxation mechanism.

Electron Energy Loss Spectroscopy (EELS) of the NWs is used to obtain the elemental composition of the NW. In particular, relative elemental quantification of the In vs Ga ratio in atomic % is shown in Fig. S2a and b respectively. Ga diffusion towards the InAs channel of 8-10 % is observed along the $[0\,0\,1]$ direction. The diffusion region is highlighted in Fig. S2a between the white dashed lines and is better visible in Fig. S2b. Such preferential diffusion has been reported in GaAs(Sb)/InGaAs/InAs NWs grown on GaAs(0 0 1) substrates[2]. It has been proposed that Ga diffusion arises as a thermally activated strain minimization mechanism during growth of lattice-mismatched InAs and GaAs. It should be noted that an In rich region only exists at the outermost layers of the conduction channel whereas the region in between is diluted with the diffused Ga. This can be circumvented by further reducing the substrate temperature during InAs growth, but is not attempted because it comes at the cost of abating the selectivity of the sample.

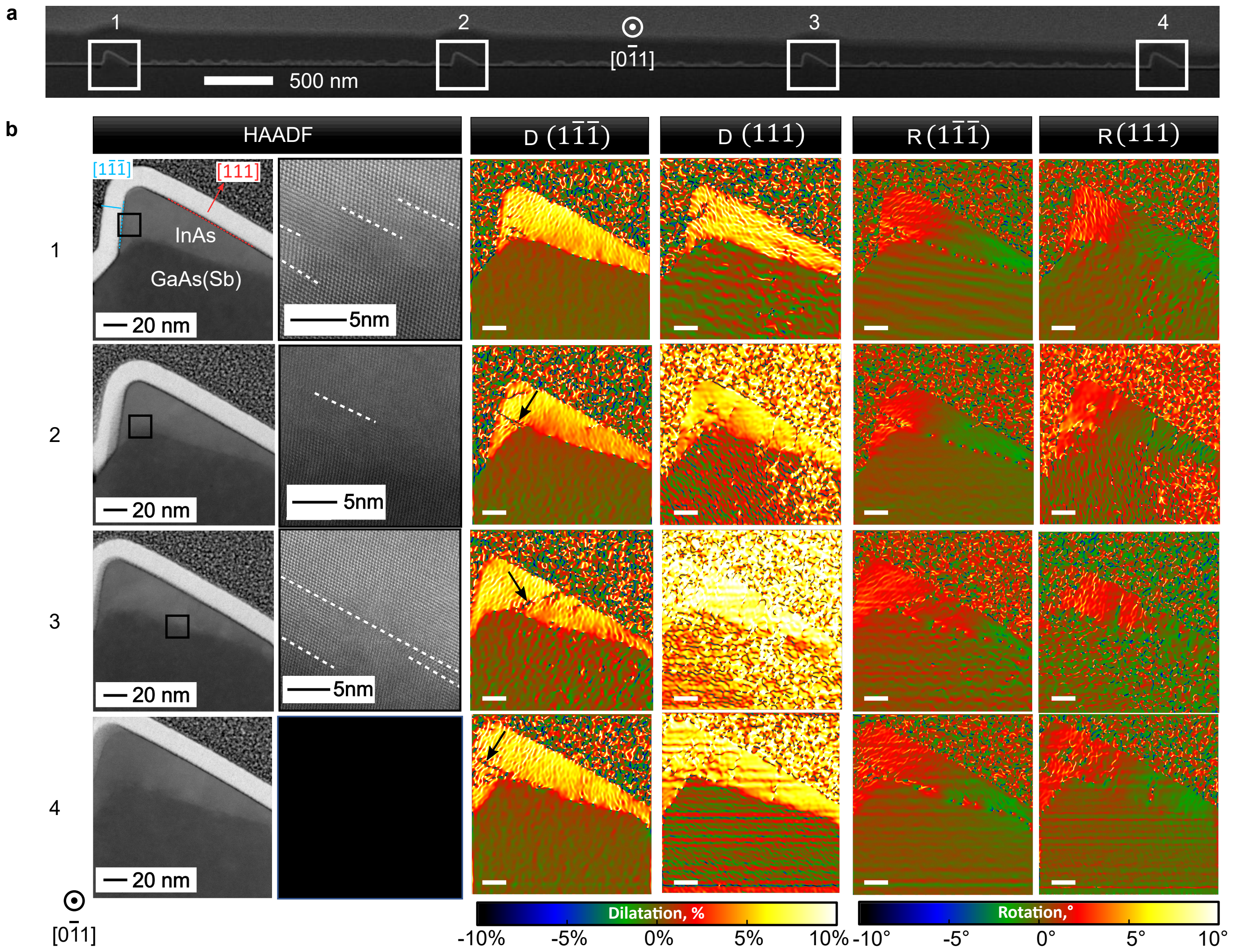

FIG. S1. Cross-section HRTEM and GPA analysis of the NWs used in the measurements. **a** A cross-sectional view of 4 InAs/GaAs(Sb) NWs roughly from the middle of an array of 50 NWs oriented along the [0 $\bar{1}$ 1] direction on a GaAs(3 1 1)A substrate. **b** (column 1) High-angle annular dark field scanning tunneling micrographs (HAADF-STEM) show the GaAs(Sb) and InAs regions of the NWs. The morphology of all 4 NWs is uniform and the InAs channel exhibits {1 1 1}A facets. **b** (column 2) Stacking faults originating at the GaAs(Sb)/InAs interface are highlighted with white dashed lines. **b** (column 3, 4) Dilatation maps of all NWs show an array of dislocations at the GaAs(Sb)/InAs interface, and arrows point to stacking faults. **b** (column 5, 6) The rotational maps indicate rotation of the crystal planes between the GaAs(Sb) buffer and the InAs channel. Scale bars correspond to 20 nm in all dilatation and rotation maps.

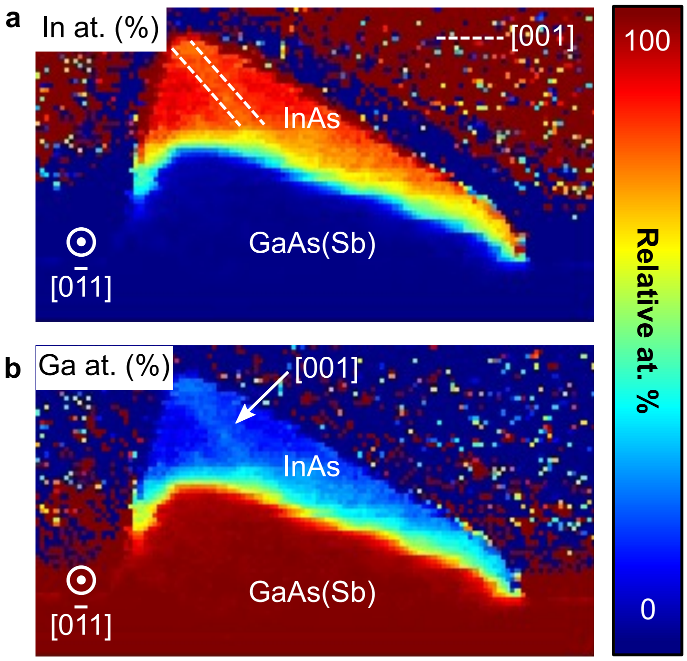

FIG. S2. EELS composition maps of the NW cross-section. **a** In and **b** Ga relative atomic % compositional map of a typical NW used in this study obtained by electron energy loss spectroscopy (EELS). The InAs region is found to be diluted with $8-10\%$ Ga. The white dashed lines in **a** show the Ga-rich region within the InAs channel along which Ga is found to diffuse more than in the surrounding region. The preferential diffusion of Ga along the [0 0 1] direction is better visible in **b** where the map is relative to the Ga atomic %.

S3. ELECTRICAL CHARACTERIZATION OF SAG NANOWIRE FETS.

Figure S3 shows measurements of the conductance vs. gate potential for 5 NW FETs similar to those discussed in Fig. 2 of the main manuscript. Here, only a single NW is measured ($M = 1$) and 5 consecutive wires of the SAG array were chosen. The channel length was $1\,\mu$m and measurements were performed at 100 K. In each case the conductance, $G(V_G)$, was fitted to the model of Ref.[3], $G(V_G)^{-1} = R_S + L^2/(\mu C(V_G - V_{TH}))$ where R_S, μ, V_{TH} and C are the contact/series resistance, mobility, threshold voltage and gate-NW capacitance respectively. We take $C = 5.3$fF corresponding to the value extracted from the simulation described in Section S4. The resulting mobility and threshold voltage for each device is stated in the figure. The mobility is between 56 and 140 cm^2/Vs and V_{TH} is between -0.63 V and -0.93 V consistent with the discussion in the main text of un-gated FETs not being in pinch-off. The values are relatively low for InAs which we attribute to the stacking faults and dislocations associated with the unbuffered growth of InAs on non-lattice matched GaAs[4].

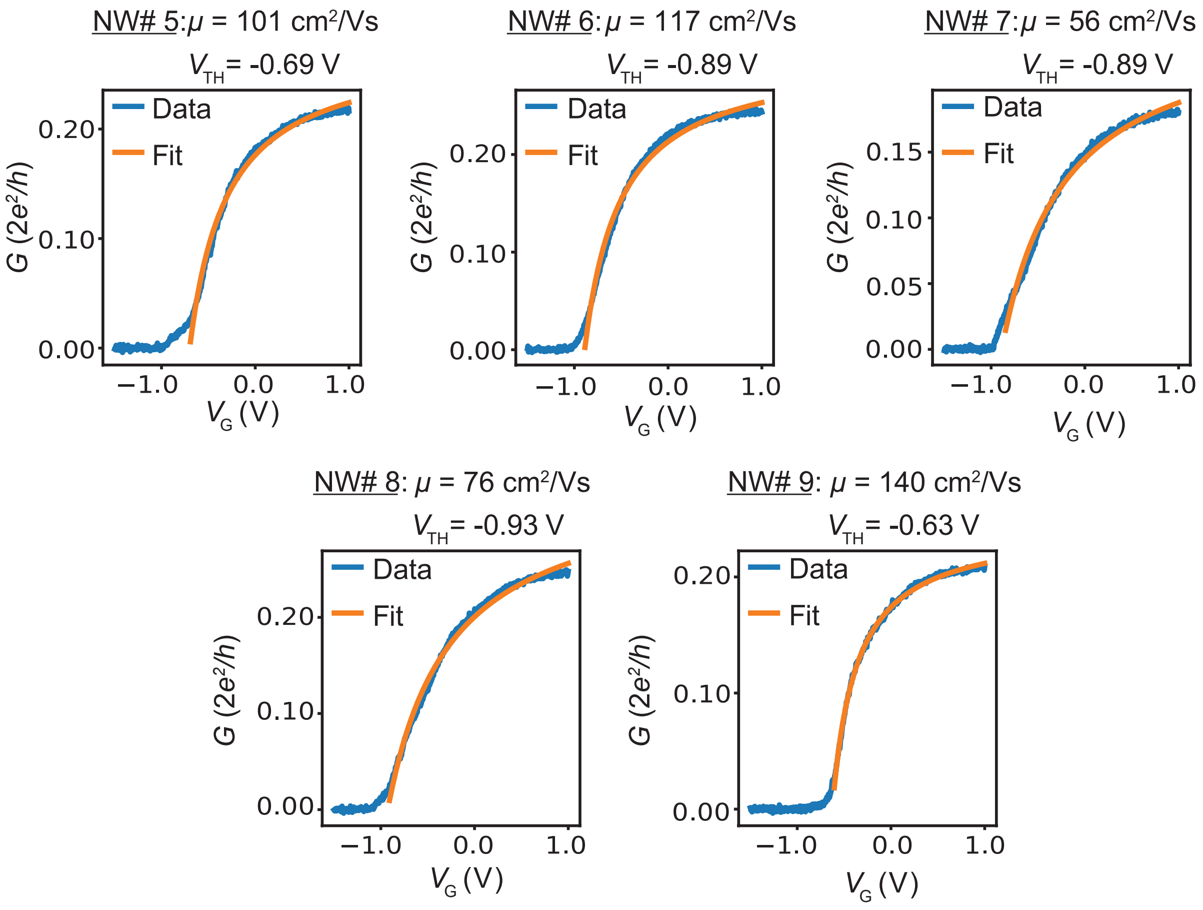

FIG. S3. Examples of fitting transistor data. Conductance vs. gate potential for 5 SAG nanowire FETs. The devices are similar to Fig. 2 of the main manuscript and are fabricated on 5 consecutive single SAG NWs of the arrays. The measurements were performed at 100 K and the parameters extracted from the fit are included above in each case.

S4. MODELLING GATE CAPACITANCE OF NWFETS

Gate capacitance for NWFET devices is estimated using the ANSYS® Electronics Desktop 2021 R2 software. Model NWFET devices are rendered based on measurements from AFM, SEM, and cross-section TEM for lengths 50 nm, 300 nm, 800 nm, 3 μm, and 6 μm of the semiconductor NW segment. An example model is shown in Fig. S4a. The Maxwell3D module of the software models electrostatics using finite element analysis and solves Maxwell's equations in a finite region of space. From the resulting values shown in Fig. S4b a capacitance per unit length of $\sim 5.3\,\mathrm{fF}/\mu\mathrm{m}$ is extracted to find the capacitance for other NW segment lengths.

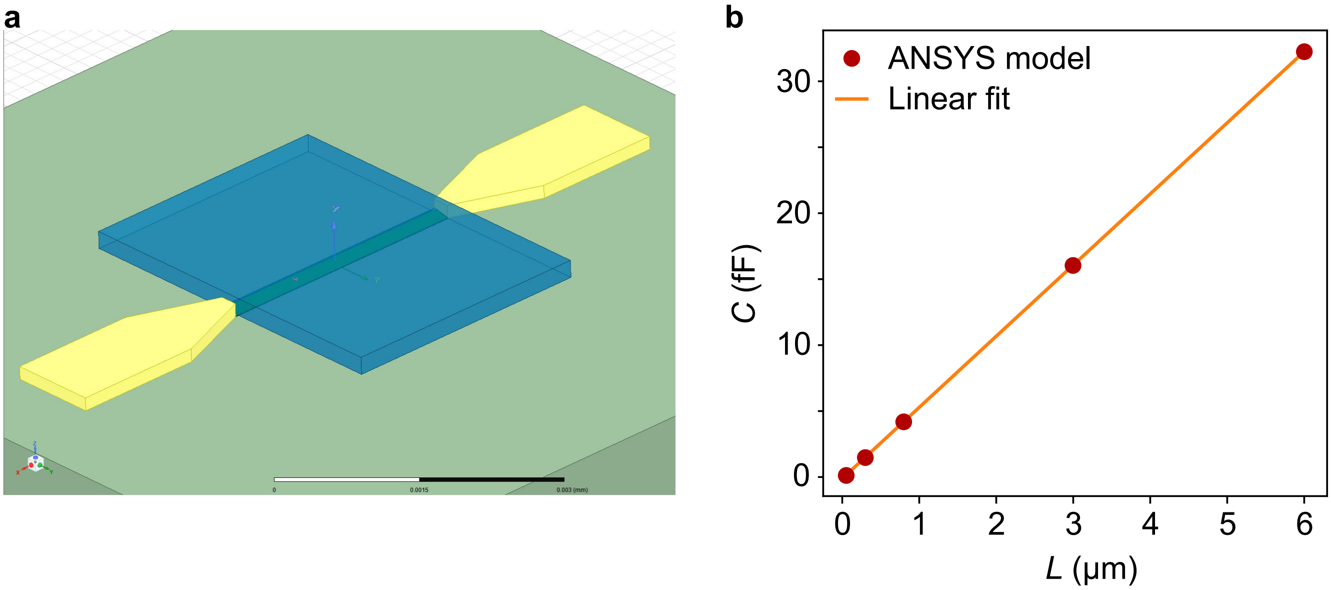

FIG. S4. ANSYS model of gate capacitance in NWFETs of varying channel length. **a** 3D model of a NWFET device with a 3 μm long InAs channel rendered in ANSYS software based on AFM, SEM, and TEM measurements. Ti/Au contacts in yellow, Ti/Au topgate in blue **b** Capacitance values calculated by the model for different InAs channel lengths and linear fit to the results.

S5. DIAGONAL OF THE CONDUCTANCE MATRIX WITH $V_{\mathrm{G}} = -1\,\mathrm{V}$

Figure S5a,b shows the conductance along the diagonal of the conductance matrix of Fig. 3c for MUX/d-MUX channels #300 to #398 for $V_{\mathrm{G}} = 1$ V and $V_{\mathrm{G}} = -1\,\mathrm{V}$, respectively. Odd channels addresses NW SAG FETS at the DUT level while even channels are shorted. The shorted channels are unaffected by V_{G} while the FETs have $G = 0\,2e^2/h$ at negative V_{G}. For the conductance matrix in Fig. 3c of the main manuscript, this corresponds to every second pixel along the diagonal showing no conductance as discussed in the main manuscript.

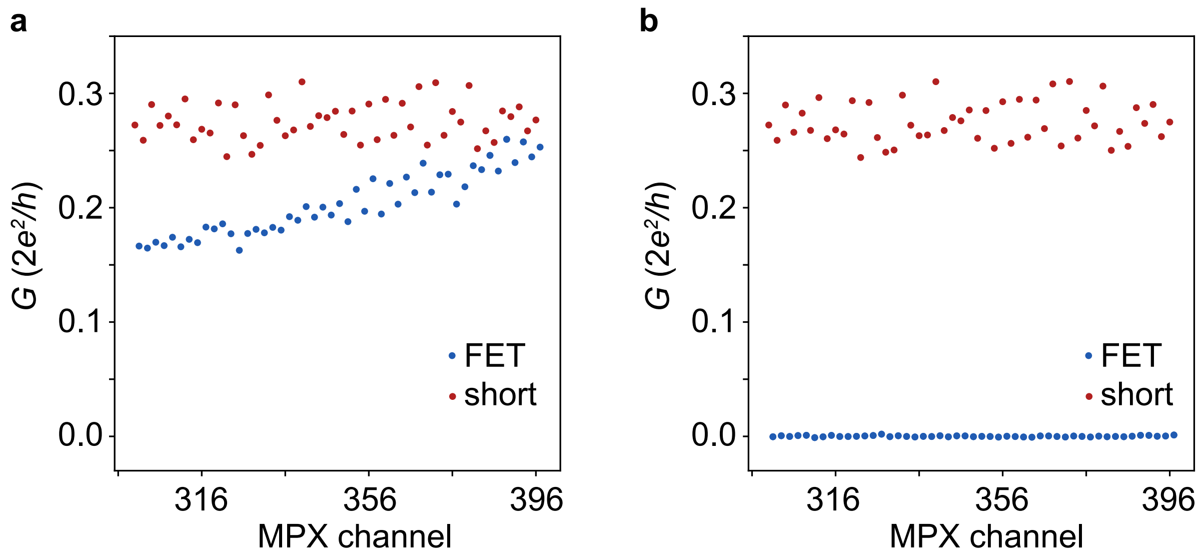

FIG. S5. Verification of DUT layer function. **a** (**b**) Conductance of the MUX/d-MUX circuit when both MUX and d-MUX addresses the same channels #300 to #398 with $V_{\mathrm{G}} = +1\,\mathrm{V}$ ($V_{\mathrm{G}} = -1\,\mathrm{V}$). Odd channels addresses NW FETS at the DUT level while even channels are shorted.

S6. ADDITIONAL MULTIPLEXER CIRCUITS

Other multiplexer circuits were created in the scope of the research presented here. Fig. S6 shows stitched optical microscope micrographs and the corresponding conductance matrices of three additional MUX/d-MUX circuits fabricated on another chip from the same nanowire growth as the one shown in the main text. All three designs are fully functional and have 16, 64, and 128 outputs respectively. A broken gate line (inset in Fig. S6a) is the cause for the off-diagonal features in the conductance matrices in agreement with simulations described in S7.

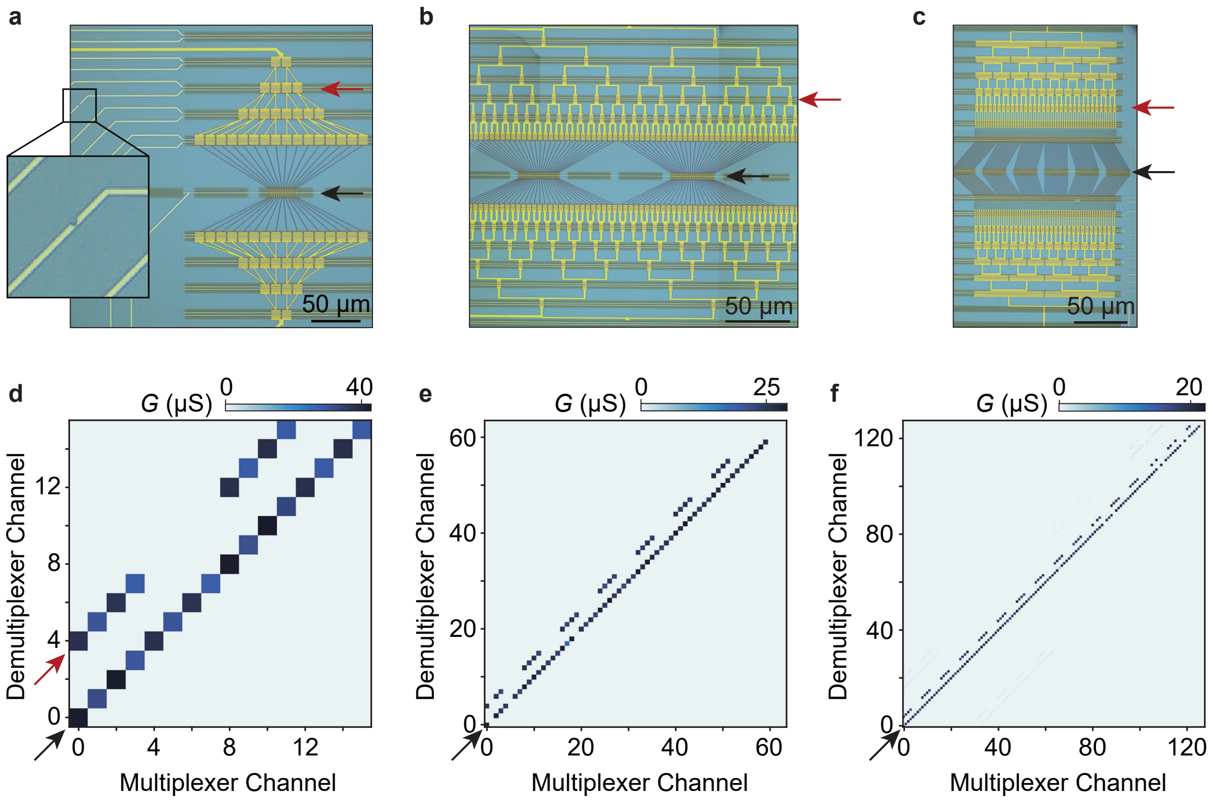

FIG. S6. Examples of additional multiplexer circuits. **a-c** stitched optical microscope micrographs of three MUX/d-MUX circuits with 16, 64, and 128 outputs respectively. Red arrows indicate the level responsible for the off-diagonal features in d-f. **d-f** the corresponding conductance matrices.

S7. CONDUCTANCE MATRIX SIMULATIONS

To verify that the patterns observed in the conductance matrices agree with the assumed gate failures a Monte Carlo simulation was constructed. The simulation takes the number of levels in the multiplexer L as an input and gate failures on both the MUX and d-MUX side can be specified. The simulation assumes that a failing gate is non-functional across the entire level. The algorithm then simulates a random walk from source to drain of $2^L \times 10$ walkers for all MUX and d-MUX channel combinations. If a walker encounters a FET in depletion it is discarded, if at least one walker reaches the end of the circuit, a value of 1 is assigned to the corresponding matrix element. Figure S7 shows simulated conductance matrices of the circuits shown in Fig. S6 (Fig. S7 a-c) and main text Fig. 3 (Fig. S7 d). For the simulations in Fig. S7a-c it was specified that the gate highlighted in the inset of Fig. S6a is broken and for the simulation in Fig. S7d it was specified that gates are broken in levels 0 and 6 in the MUX side and on level 7 in the d-MUX side. However, it must be noted that identical matrices can result from different combinations of broken gates.

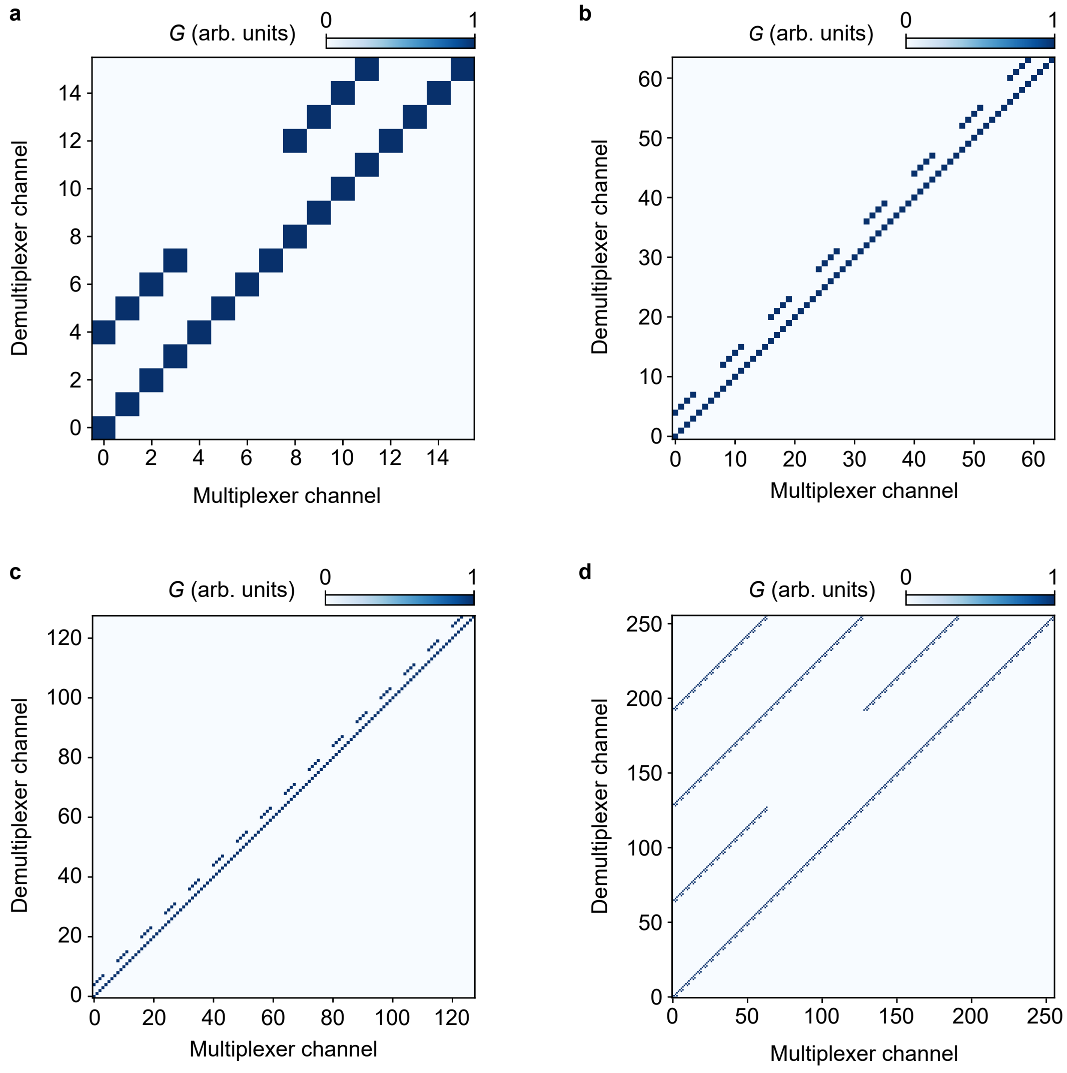

FIG. S7. Simulations of gate failures reproducing observed data. **a-c** Simulated conductance matrices for the circuits shown in Fig. S6. The gate identified in the inset in Fig. S6 is assumed to be broken. **d** Simulated conductance matrix for the circuit in main text Fig. 3. Gate failures chosen to match the observed matrix pattern.

S8. EXAMPLES OF CONDUCTANCE MATRICES OF MULTIPLEXERS CONTAINING MULTIPLE FAULTY FET GATES

In Fig. 3e,f of the main manuscript, the consequences for the conductance matrix of individual FETs or FET rows failing to pinch-off at various locations of the MUX/d-MUX circuit were discussed. When multiple FETs or FET rows fail at the same time, their consequences for the conductance matrix do not just add but are coupled. As an example, the purple arrow in Fig. 3c of the main manuscript, appears for multiplexer channel 0 and demultiplexer 192. Since 192 is not a power of 2, such a feature cannot be related to the failing of a FET row. Rather, 192=128+64 and is a consequence of the combined faults located at row 6 ($64 = 2^6$) and 7 ($128 = 2^7$) (the FETs closest to the DUT layers is nr. 0). To illustrate such coupling explicitly, Fig. S8a-e shows the consequences of errors at layer 1 (periodicity 2) and 2 (periodicity 4) and their combination (periodicity 2,4 and 6). Here, the colored crosses on the schematic circuit indicate a FET failing to pinch-off (i.e. always open), and the resulting non-vanishing elements of the conductance matrix are shown below. In these schematics, the DUT layer is considered fully conducting. If faults occur on opposing branches or on opposing sides of the multiplexer circuit, the compound errors result in a more intricate pattern as shown in Fig. S8g-l.

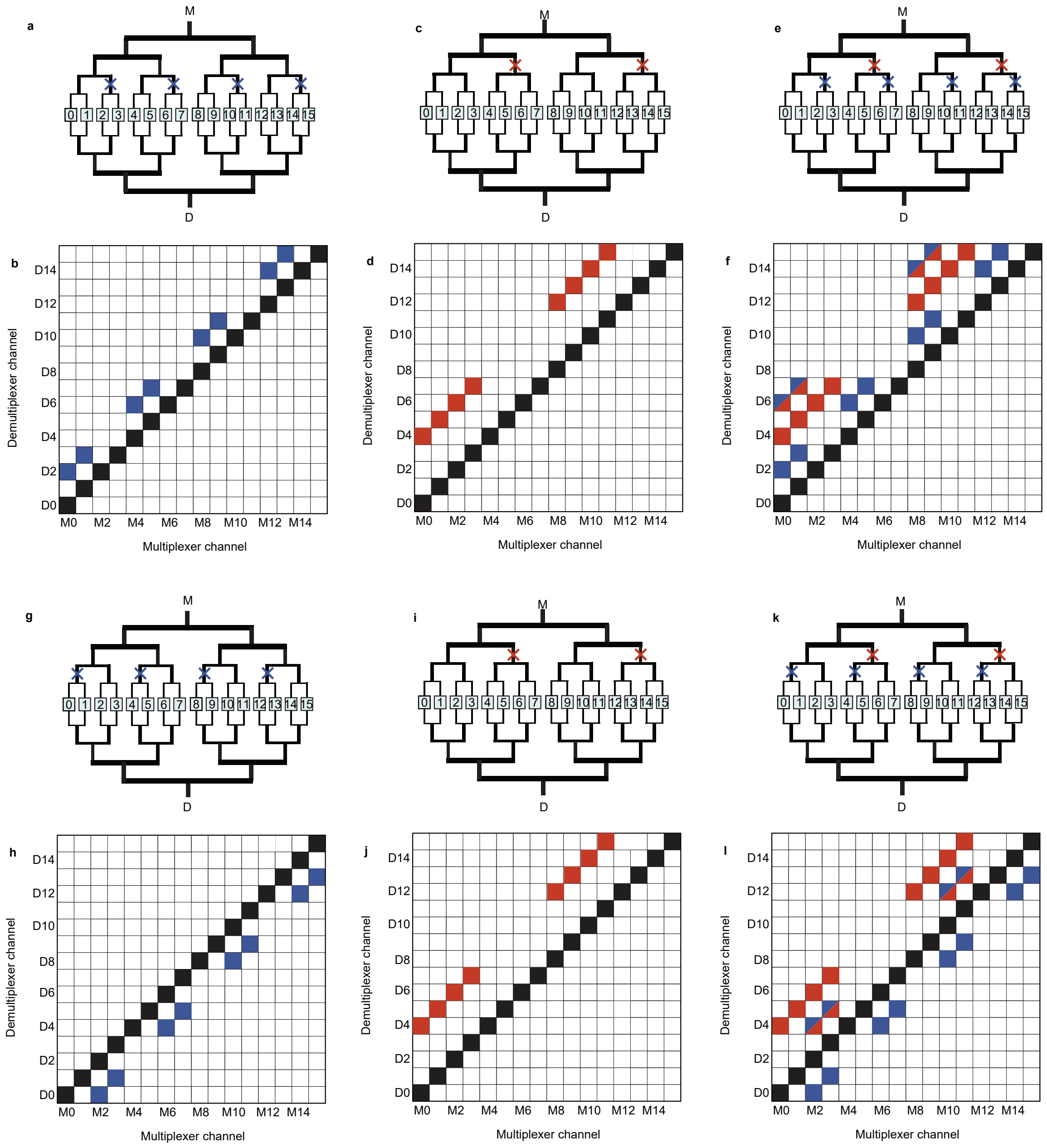

FIG. S8. Examples of compounding gate failures in conductance matrices. Examples of conductance matrices for a 16x16 multiplexer/demultiplexer circuit containing FET rows failing to pinch-off at various locations in the circuit. The examples illustrates the non-obvious consequences of multiple faults combined (panels e,f and k,l). The colors used in the matrices correspond to the colored crosses in the schematics.

S9. MEASUREMENT BANDWIDTH

All measurements were conducted in a dilution cryostat optimised for low electron temperature by including 5-stage RC and 21-stage pi-filters on each measurement line. The rejection of high-frequency radiation assists in obtaining a low electron temperature but naturally limits the measurable bandwidth to a few kHz. While this prevented us from benchmarking our InAs transistors at frequencies up to ∼ 100 GHz as previously demonstrated,[5–7] we show in Fig. S9 that MPX elements consisting of both one and 128 SAG NWs were capable of operating at frequencies up to the cut-off set by the cryostat filtering. To do this, a sinusoidal signal, $V_{\rm ac}\sin(\omega t)$ was applied to the gate of the selected MPX element, offset with a dc voltage $V_{\rm dc}$ such that the transistor was operating in a linear region of drain current $I_{\rm d}$ vs $V_{\rm dc}$ (Fig. S9a). This ensured high-fidelity transduction (Fig. S9a, inset). Fig. S9b shows the amplitude of the resulting sinusoidal $I_{\rm d}$ component plotted as a function of frequency $f = \omega/2\pi$, normalised to the amplitude at 100 Hz. Shown also is the frequency response of two cryostat lines shorted on another sample board loaded during the same cooldown. The frequency responses fall along three paths, correlated with the three different break-out boxes and looms that the lines were grouped in. This indicates that the observed 3-8 kHz low-pass cut-off is set by the cryostat filtering and wiring, with the multiplexer operating without degradation until this point.

To demonstrate the multiplexer switching capabilities within these limits, Fig. S9c shows the MPX drain current as a function of time in response to a 1 kHz, 3 V square wave applied to the gate of a single transistor in the MPX, centered around $V_{\rm g} = 0$ (Fig. S9d). The transistor switches from fully open to fully closed, with the output stable after the filter-defined ∼ 0.1 ms rise time. While this represents the upper limit of operation speed in our current set-up, future experiments are planned to measure the bandwidth of SAG devices without these limitations.

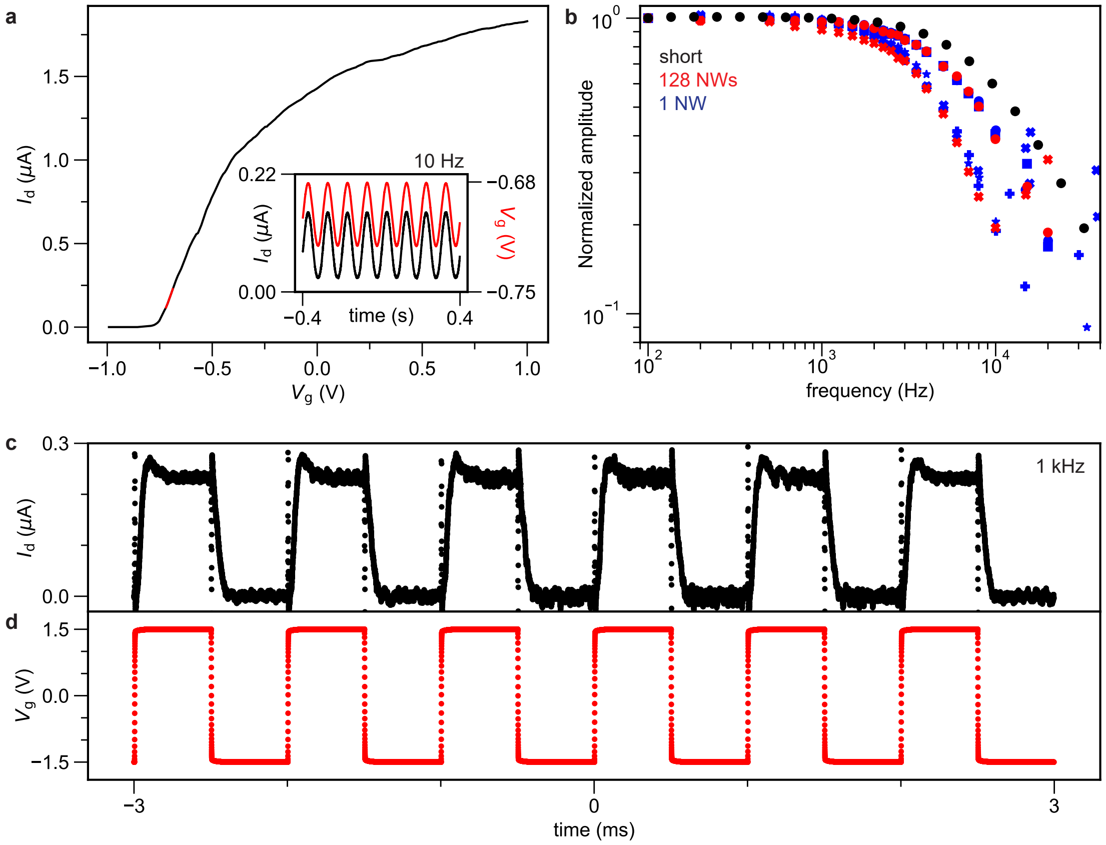

FIG. S9. Multiplexer bandwidth analysis. **a** Drain current $I_{\rm d}$ at fixed dc source-drain voltage $V_{\rm sd} = 0.1$ V in response to gate voltage $V_{\rm g}$ with no ac component. Inset: $I_{\rm d}$ modulation vs time (black) in response to $V_{\rm g} = -0.7 + 0.04\sin(20\pi t)$ V (red), i.e., an ac voltage at 10 Hz with amplitude $V_{\rm ac} = 0.04$ V, offset by $V_{\rm dc} = -0.7$ V to span the red section of the trace in the main panel. **b** Amplitude of the ac-component of $I_{\rm d}$ normalised to the amplitude at 100 Hz as a function of the frequency for two shorted cryostat lines (black), two transistors with 128 SAG NWs (red), and five transistors consisting of a single SAG NW (blue). The cut-off frequency is set by the cryostat wiring. **c** $I_{\rm d}$ response of a single SAG NW to a (d) 3 V, 1 kHz square wave applied to the gate.

S10. QD CAPACITANCES AND GATE CROSS-COUPLING

Figure S10**a** shows a zoom of a single NW with the three gates used for tuning QD in the main text and panel **b** schematically shows the couplings taken into account. The capacitance 0.37 fF from the middle gate to the NW QD was estimated by a simple parallel plate capacitor taking typical values $\varepsilon_{HfO} = 18$, oxide thickness of 15 nm, nanowire width of 180 nm, and a gate width of 200 nm. As seen in Fig. 4**c** of the main text there is negligible coupling between the gates V_T and V_B. Figure S10c shows a typical measurement of the conductance vs. V_M and V_B illustrating the ratio of couplings between the two gates to the QD formed in the middle segment and resonances attributed to QDs below the barrier gates. The couplings were characterized for an number of devices and the average values were used for compensation when acquiring the bias spectroscopy in Fig. 4**b** of the main text and for measuring $G(V_\mathrm{M})$ for the QD ensemble: $V_\mathrm{T/B} = V^0_\mathrm{T/B} + C_\mathrm{T/B,M}V_\mathrm{M}$ where $V_\mathrm{T}, V_\mathrm{B} = 140, 40\mathrm{mV}$ set the overall tuning (e.g. red mark in Fig. 4**c** the main manuscript) and the second term compensates for capacitive couplings $C_\mathrm{T,M} = -0.032$ and $C_\mathrm{B,M} = -0.022$ between the middle and top(bottom) gates. Figure S10d shows a high-resolution bias spectroscopy. An example of a discrete excited state is indicated with $\Delta E \sim 0.1E_\mathrm{C}$. This behavior is typical for all the acquired bias spectroscopy, and evolution of the addition energy with magnetic field was also consistent with an excited state spectrum with typical spacings of 10/20% of E_C.

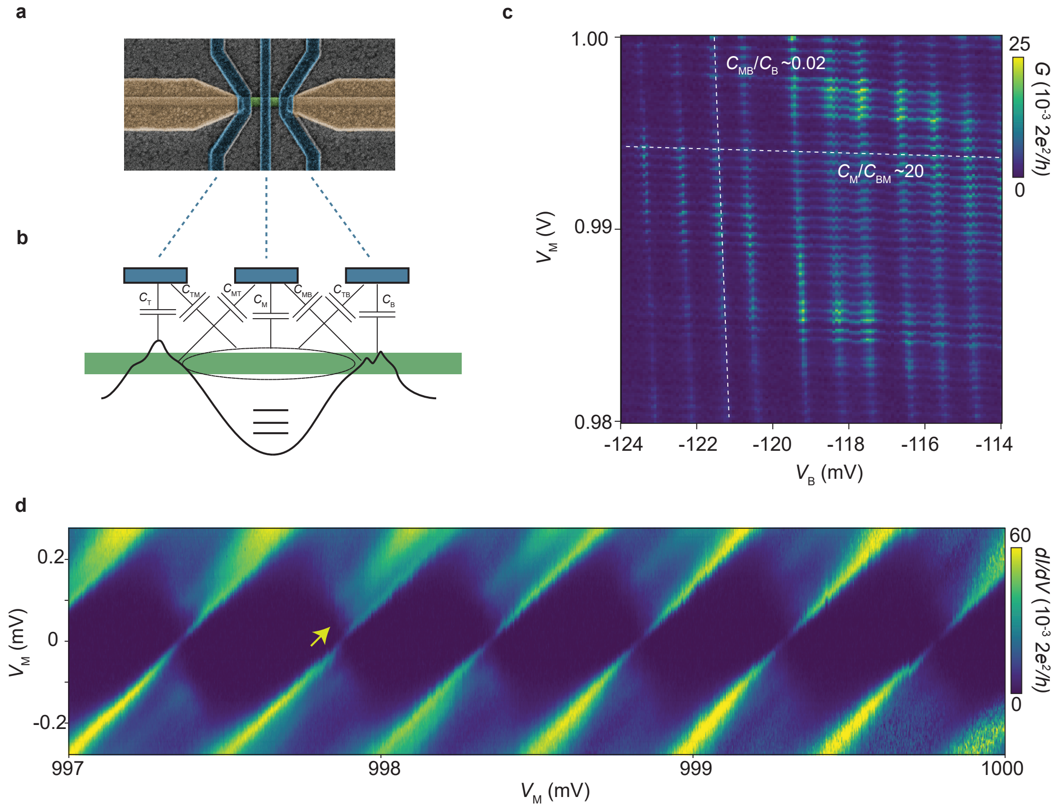

FIG. S10. Analysis of QD device gate cross-coupling. **a** SEM of a single QD device of the array. **b** Illustration of the gate cross-couplings. **c** Measurement device conductance vs. V_M and V_B. The fine structure which is nearly independent of V_V corresponds to the CB peaks of the QD in the middle segment, while vertical structures corresponds to resonances below the barrier gate. The slopes yields the ratio of gate-couplings as indicated. **d** Typical bias spectrum with indication of excited state.

S11. SOURCE-DRAIN BIAS SPECTROSCOPY ON OTHER QD DEVICES

Fig. S11a-s shows the source-drain bias spectra of the remaining 19 devices used for the analysis. All bias spectra were taken sequentially at the V_T, V_B settings as used in Fig. 4c of the main text over the same $\mathrm{V_{SD}}$ and $\mathrm{V_M}$ range. All devices exhibit Coulomb blockade physics. However, these data were taken before the results of main text Fig. 4h were compiled, and therefore not all devices were in deep Coulomb blockade at this particular gate configuration, which was chosen to optimise the behaviour of Dev1. Manually performing the analysis in main text Fig. 4h required contributions from half of the authors and took many days to complete; combining multiplexing with rapid machine-assisted analysis is the next step in simultaneously optimising device behaviour in quantum dot arrays.

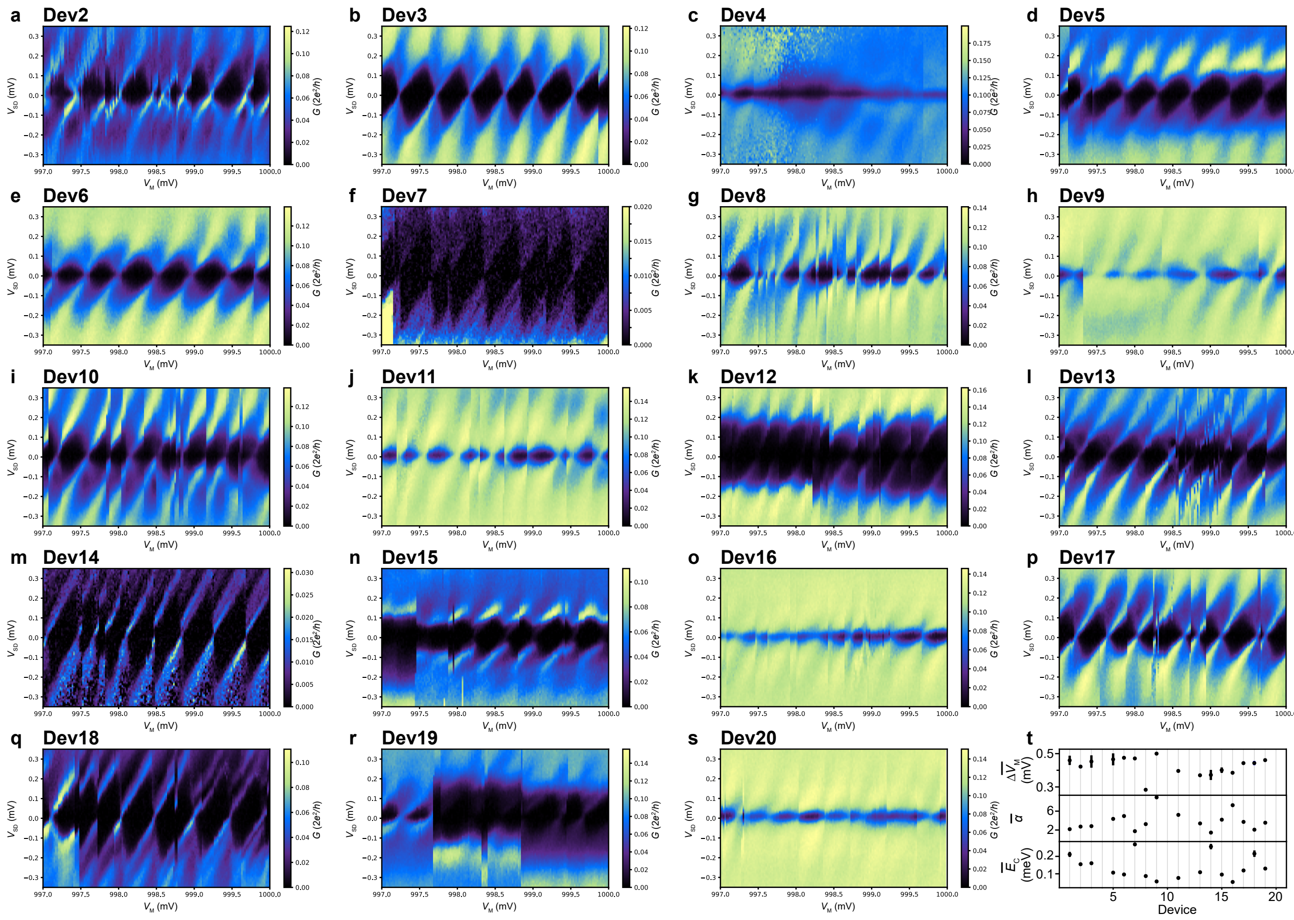

FIG. S11. Source-drain bias spectra of other QD devices. **a-s** Source-drain bias spectroscopy of other devices used for the QD analysis in main text. **t** Mean CB peak spacing $\overline{V_\mathrm{M}}$, mean charging energy $\overline{E_\mathrm{C}}$, and mean leverarm $\overline{\alpha}$ extracted for each device with at least one full well-defined Coulomb diamond in the measurements in a-s. Error bars show the standard deviation.

The height and width of each full and well-defined Coulomb diamond was manually measured to extract the mean charging energy $\overline{E_\mathrm{C}}$ and mean Coulomb peak spacing $\overline{V_\mathrm{M}}$ as well as the mean leverarm $\overline{\alpha} = \frac{\overline{V_\mathrm{M}}}{\overline{E_\mathrm{C}}}$ for each device. The resulting values are shown in Fig. S11t and the average of $\overline{E_\mathrm{C}}$ was used as an estimate of the QD charging energy in the analysis in the main text.

S12. EXAMPLES OF CONDUCTANCE AS A FUNCTION OF V_{M}

Figure S12a shows examples of $G(V_{\mathrm{M}})$ normalized 0 to 1 for all devices with $V_{\mathrm{T}} = -50\,\mathrm{mV}$, $V_{\mathrm{B}} = -50\,\mathrm{mV}$ to $-200\,\mathrm{mV}$, equivalent to the rightmost column of main text Fig. 4h. The data and fit for Dev1 with $V_{\mathrm{T}} = -50\,\mathrm{mV}$, $V_{\mathrm{B}} = -50\,\mathrm{mV}$ is shown in Fig. S12b.

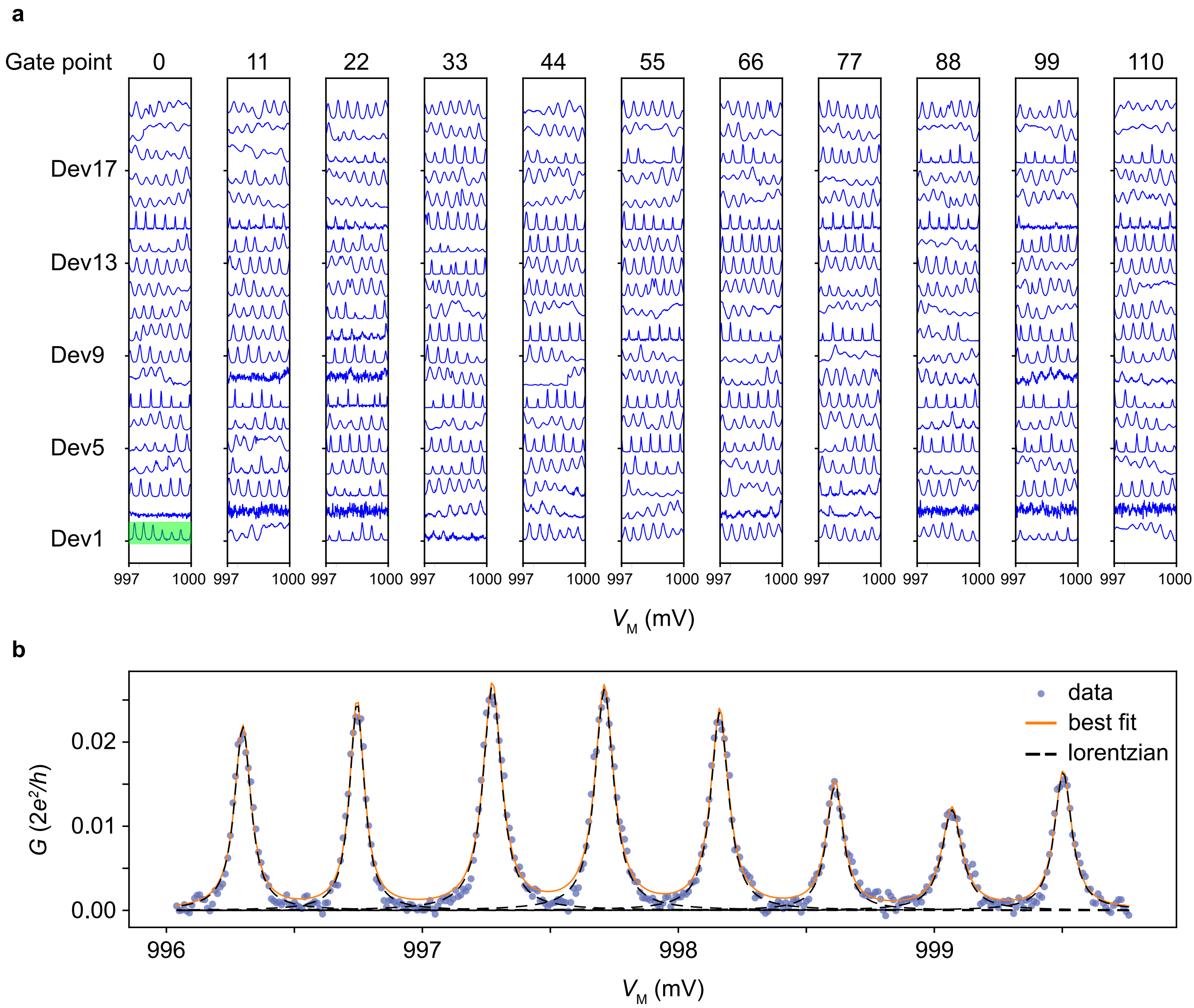

FIG. S12. Examples of Coulomb physics in Dev1-Dev20. **a** Examples of $G(V_{\mathrm{M}})$ normalized 0 to 1 in conductance and offset by 1.2 for gate points corresponding to the rightmost column of main text Fig. 4h ($V_{\mathrm{T}} = -50\,\mathrm{mV}$) **b** The raw data (blue circles) of Dev1 with $V_{\mathrm{T}} = -50\,\mathrm{mV}$, $V_{\mathrm{B}} = -50\,\mathrm{mV}$ (green box in a) with best fit (orange line) and individual lorentzians (black dashed lines).

S13. DEV1 $G(V_{\mathrm{M}})$ FOR ALL V_{T} AND V_{T}

The data presented in Fig. S13 shows $G(V_{\mathrm{M}})$ for all V_{T} and V_{B} for Dev1 normalized 0 to 1 in conductance and offset by 1.2 for clarity. The non-normalized data was used for ΔV_{M} shown in main text Fig. 4d

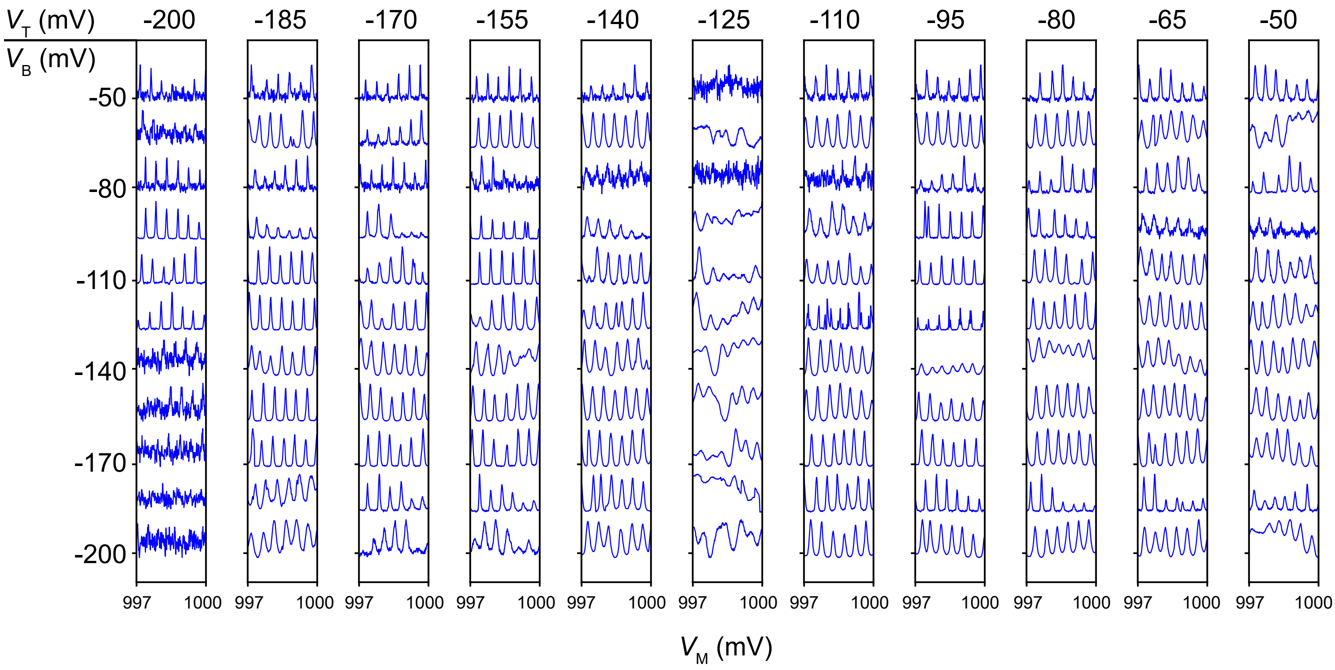

FIG. S13. Dev1 behaviour at all examined barrier gate tunings. $G(V_{\mathrm{M}})$ normalized 0 to 1 in conductance and offset by 1.2 for all V_{T} and V_{B} for Dev1.

S14. HISTOGRAMS OF Γ AND $\overline{\Delta V_{\mathrm{M}}}$ FOR INDIVIDUAL DEVICES

Figure S14 shows histograms of mean tunnel coupling $\overline{\Gamma}$ and Coulomb peak spacing $\overline{V_{\mathrm{M}}}$ for each individual QD device illustrating the overall device to device variance. The red line shows the overall mean across all devices.

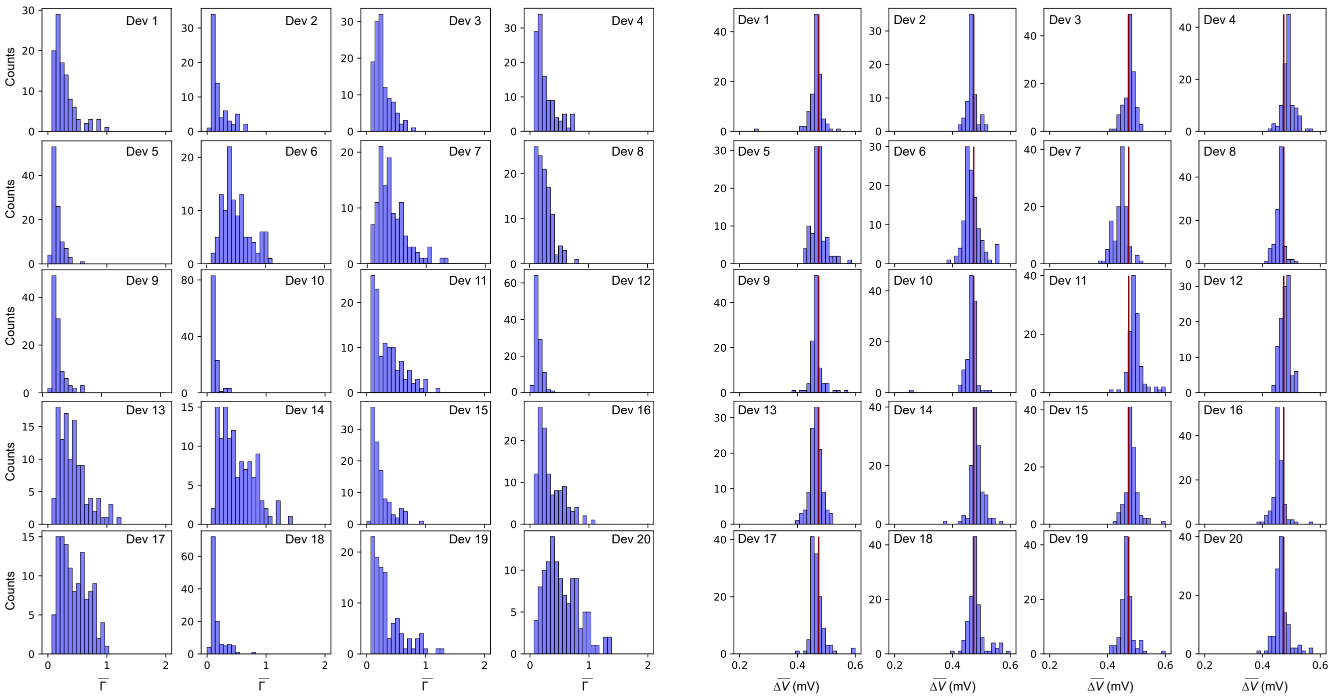

FIG. S14. Histograms of $\overline{\Gamma}$ and $\overline{\Delta V_{\mathrm{M}}}$ for the individual devices. The red line in the $\overline{\Delta V_{\mathrm{M}}}$ histograms shows the overall mean across all devices.

SUPPLEMENTARY REFERENCES

[1] Isu, T., Hata, M., Watanabe, A. & Katayama, Y. In situ scanning microprobe reflection high-energy electron diffraction observation of GaAs surfaces during molecular-beam epitaxial growth. *J. Vac. Sci. Technol., B* **7**, 714 (1998).

[2] Beznasyuk, D. V. *et al.* Doubling the mobility of InAs/InGaAs selective area grown nanowires. *Phys. Rev. Mater.* **6**, 034602 (2022).

[3] Gül, Ö. *et al.* Towards high mobility InSb nanowire devices. *Nanotechnology* **26**, 215202 (2015).

[4] Beznasyuk, D. V. *et al.* Doubling the Mobility of InAs/InGaAs Selective Area Grown Nanowires. *Phys. Rev. Materials* **6**, 034602 (2022).

[5] Tomioka, K., Yoshimura, M. & Fukui, T. A III–V Nanowire Channel on Silicon for High-Performance Vertical Transistors. *Nature* **488**, 189–192 (2012).

[6] Egard, M. *et al.* Vertical InAs Nanowire Wrap Gate Transistors with $F_t > 7$ GHz and $F_{max} > 20$ GHz. *Nano Letters* **10**, 809–812 (2010).

[7] Johansson, S., Memisevic, E., Wernersson, L.-E. & Lind, E. High-Frequency Gate-All-Around Vertical InAs Nanowire MOSFETs on Si Substrates. *IEEE Electron Device Lett.* **35**, 518–520 (2014).
